# Determination of the complete mitochondrial genome of *Lardoglyphus konoi* (Acari: Astigmata)

**DOI:** 10.1080/23802359.2021.1990147

**Published:** 2021-10-15

**Authors:** Mei-Qing Wang, Run Yao, Feng Jiang, Xiao-Dong Zhan

**Affiliations:** Department of Medical Parasitology, Wannan Medical College, Wuhu, Anhui, China

**Keywords:** *Lardoglyphus konoi*, mitochondrial genome, mites, truncated tRNA

## Abstract

The mitochondrial genome of *Lardoglyphus konoi* was 14,269 bp long and consisted of 37 genes including 22 transfer RNAs, 13 protein-coding genes and 2 ribosomal RNA genes. All the 13 protein-coding genes had complete start/stop codons. Most inferred tRNAs were extremely truncated and the T- or D-arm was missing. The position of two rRNAs and the largest non-coding region were separated by tRNAs. The Bayesian phylogenetic tree indicated that *L. konoi* was closely related to *Carpoglyphus lactis.*

*Lardoglyphus konoi* (Acari: Lardoglyphidae) (Sasa and Asanuma 1951) belongs to the family Lardoglyphidae. Generally, adults and nymphs of *Lardoglyphus* feed on drying or dried fish (Olsen 1982). Studies have reported the internal morphology and biological behaviors of *L. konoi* (Okamoto et al. 1991). However, the genetic characteristics of *L. konoi* has not been reported.

In the present study, *L. konoi* were collected from the Lugang Market, Yijiang District, Wuhu City (118°21′11ʺE, 31°20′16ʺN), Anhui Province, China, at 22 September 2020, and then cultured with dried fish as food in the laboratory. A specimen was deposited at the Wannan Medical College (Xiao-Dong Zhan, xdzhan@126.com) under the specimen number (Lk 20200109). Total genomic DNA was extracted using a DNeasy Blood & Tissue Kit (QIANGEN GmbH, Germany). The extracted DNA was fragmented using a Covaris S220-DNA Shearing machine (Thermo), and 500-bp fragments were recovered using Hieff NGS DNA Selection Beads (Yeasen, Shanghai, China). The sequencing library was prepared using a NEB Next Ultra DNA Library Prep Kit (NEB, England) and then sequenced by the Sangon Biotech Co. Ltd., (Shanghai, China) using an Illumina Hiseq sequencer. The mitogenome was assembled using NOVOPlasty v4.1 (parameters: K-mer = 39; Genome range = 120,000–220,000; Dierckxsens et al. 2017) and annotated using MITOS2 WebServer with default parameters (Bernt et al. 2013).

The complete mitochondrial genome (GenBank: MW784238) length of *L. konoi* was 14,269 bp, and contained 13 protein-coding genes (PCGs), 22 transfer RNAs (tRNAs), and 2 ribosomal RNAs (rRNAs). Except for *nad6* gene, ATN was used as the start codon (N means any nucleotide) in all the other PCGs. All the 13 PCGs included a complete translation termination codon, either TAG or TAA. Most of the tRNAs were atypical, lacking either the D- or T-arms. Two rRNAs *rrnS* and *rrnL* were 666 and 1031 bp in length, respectively. The largest non-coding region (292 bp) localized to the interval between *trnF* and *trnS1*.

*Lardoglyphus konoi* and 12 published mitochondrial sequences of other species were downloaded from the GenBank. The PCGs of these 13 species were aligned using the software MEGA 7 (Kumar et al. 2016), and then used to construct the Bayesian phylogenetic tree with *Phyllocoptes taishanensis* as an outgroup using the MrBayes v3.2.7 (Ronquist and Huelsenbeck 2003). The parameters for phylogenetic analysis were as follows: GTR+Ι+Γ model, ngen = 1,000,000, printfreq = 1000, samplefreq = 1000, nchains = 4. The obtained tree was viewed using FigTree (Rambaut 2014), showing that *L. konoi* was closely related to *Carpoglyphus lactis* (Figure 1).

**Figure 1.**
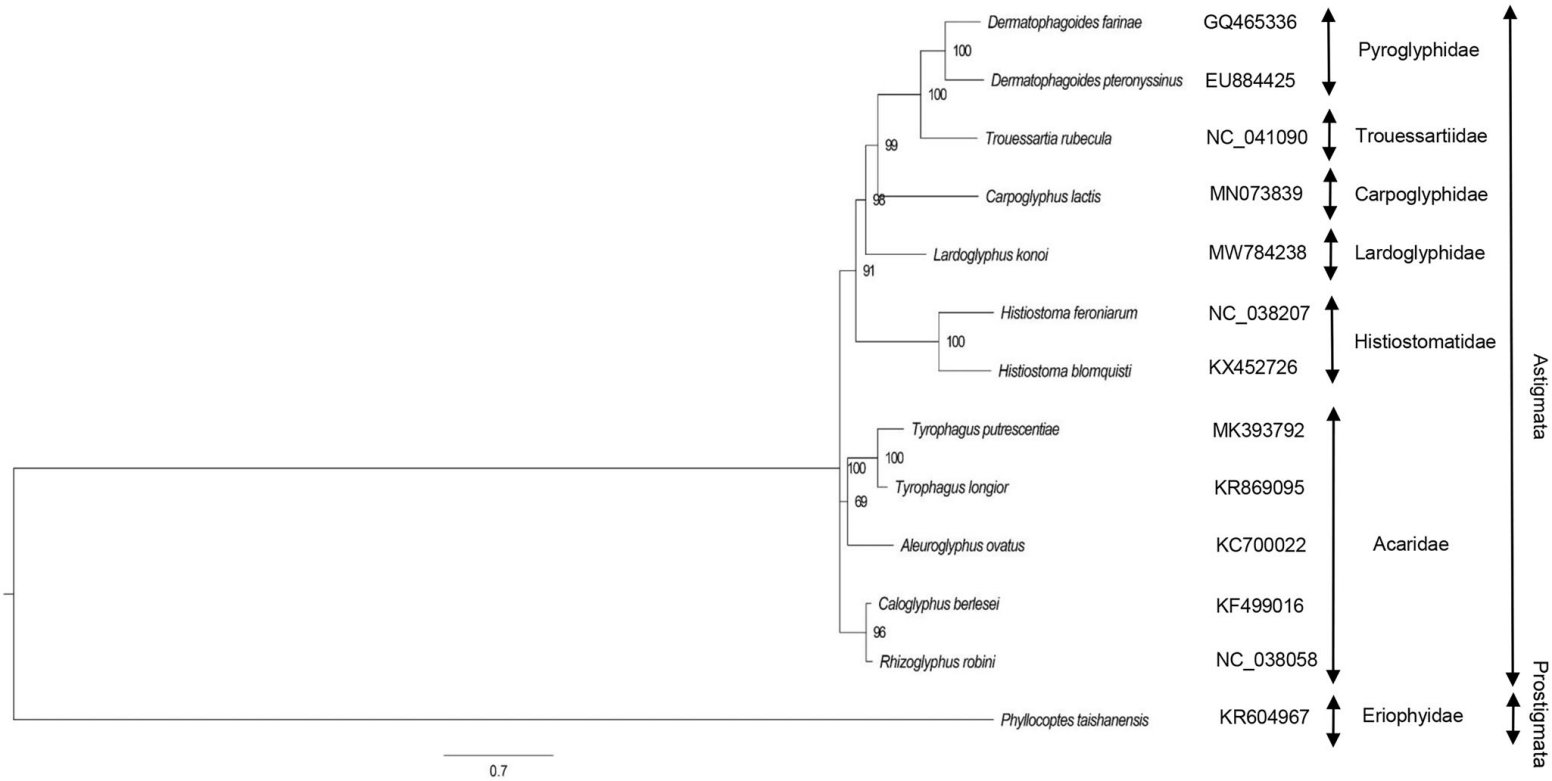
Bayesian inference tree of 11 Astigmata species and an outgroup based on 13 protein-coding genes from mitochondrial genomes. Numbers above branches indicate bootstrap values for 1000 replicates.

## Data Availability

The genome sequence data are openly available in GenBank of NCBI at https://www.ncbi.nlm.nih.gov under the accession no. MW784238. The associated BioProject, SRA, and Bio-Sample numbers are PRJNA735603, SRR14751375, and SAMN19589915 respectively.
